# Clinical characteristics and disease outcomes in ER+ breast cancer: a comparison between HER2+ patients treated with trastuzumab and HER2- patients

**DOI:** 10.1186/s12885-021-08555-4

**Published:** 2021-07-13

**Authors:** Shuai Li, Jiayi Wu, Ou Huang, Jianrong He, Li Zhu, Weiguo Chen, Yafen Li, Xiaosong Chen, Kunwei Shen

**Affiliations:** General Surgery Department, Comprehensive Breast Health Center, Ruijin Hospital, Shanghai Jiaotong University School of Medicine, 22nd Floor, 197 Ruijin Er Road, Shanghai, 200025 China

**Keywords:** Breast cancer, Hormone receptor, HER2, Trastuzumab, Endocrine therapy, Escalation

## Abstract

**Background:**

Trastuzumab has changed the prognosis of HER2+ breast cancer. We aimed to investigate the prognosis of ER+/HER2+ patients treated with trastuzumab, thus to guide escalation endocrine treatment in ER+ breast cancer.

**Methods:**

ER-positive early breast cancer patients operated at Ruijin Hospital between Jan. 2009 and Dec. 2017 were retrospectively included. Eligible patients were grouped as HER2-negative (HER2-neg) or HER2-positive with trastuzumab treatment (HER2-pos-T). Kaplan-Meier analysis and Cox proportional hazards model were used to compare the disease-free survival (DFS) and overall survival (OS) between these two groups.

**Results:**

A total of 3761 patients were enrolled: 3313 in the HER2-neg group and 448 in the HER2-pos-T group. Patients in the HER2-pos-T group were associated with pre/peri-menopause, higher histological grade, LVI, higher Ki-67 level, lower ER and PR levels (all *P* <  0.05). At a median follow-up of 62 months, 443 DFS events and 191 deaths were observed. The estimated 5-year DFS rate was 89.7% in the HER2-neg group and 90.2% in the HER2-pos-T group (*P* = 0.185), respectively. Multivariable analysis demonstrated that patients in the HER2-pos-T group had a better DFS than patients in the HER2-neg group (HR 0.52, 95% CI: 0.37–0.73, *P* <  0.001). The estimated 5-year OS rates were 96.0% and 96.3% in the two groups, respectively (*P* = 0.133). Multivariate analysis found that HER2-pos-T group was still associated with significantly better OS compared with the HER2-neg group (HR 0.38, 95% CI: 0.22–0.67, *P* = 0.037).

**Conclusion:**

ER+/HER2+ breast cancer patients treated with trastuzumab were associated with superior outcome compared with ER+/HER2- patients, indicating HER2-positivity itself may not be an adverse factor for ER+ patients in the era of trastuzumab.

**Supplementary Information:**

The online version contains supplementary material available at 10.1186/s12885-021-08555-4.

## Introduction

Breast cancer is the most common cancer and the leading cause of death in women. More than 2,088,849 new cases of breast cancer were estimated worldwide in 2018 [1]. Breast cancer can be categorized into at least four subtypes based on the status of molecular markers for estrogen receptor (ER), progesterone receptor (PR), and human epidermal growth factor 2 (HER2) [2].

About 15–20% of patients with breast cancer have HER2-amplfied and/or overexpressed tumors and about half HER2+ tumors express hormone receptor (HR) [3]. Previous reports showed that HER2 positivity was associated with a worse prognosis before the ear of anti-HER2 therapy [4–6]. In view of that, clinicians tended to recommend escalation endocrine therapy for patients with HR+/HER2+ disease, such as ovarian function suppression (OFS) in pre-menopausal women.

Clinical trials have demonstrated that addition of trastuzumab to standard chemotherapy in patients with HER2+ early-stage breast cancer significantly improved disease outcome [7–11], which was not influenced by HR status. Then, should ER+/HER2+ still be considered as an indicator of poor prognosis in the era of anti-HER2 treatment? In current study, we aimed to investigate the prognosis of ER+/HER2+ breast cancer patients treated with trastuzumab compared with ER+/HER2- patients, thus to guide us to select appropriate endocrine treatment decision making.

## Materials and methods

### Patient

Women who underwent surgery at General Surgery Department, Comprehensive Breast Health Center, Ruijin Hospital, Shanghai Jiaotong University School of Medicine from January 2009 to December 2017 were retrospectively enrolled. Clinicopathological, treatment, and follow up information were retrieved from Shanghai Jiaotong University Breast Cancer Database (SJTU-BCDB). Patients who met the following criteria were enrolled for further analysis: (1) female gender, (2) invasive breast cancer, (3) no history of prior malignancy, (4) no evidence of distant metastasis at diagnosis, (5) ER+/HER2- breast cancer or ER+/HER2+ tumors treated with trastuzumab, and (6) complete clinical and follow-up data. Those who were diagnosed with ER+/HER2+ breast cancer but received no trastuzumab treatment were excluded. This study was reviewed and approved by the independent Ethical Committees of Ruijin Hospital, Shanghai Jiao Tong University School of Medicine.

### Histopathological assessments

Tumor histo-pathologic assessment was conducted by Department of Pathology, Ruijin Hospital, Shanghai Jiaotong University School of Medicine. The methods and criteria for immunohistochemistry (IHC) assessment of ER, PR, HER2 and Ki-67 were as described in our previous reports [12, 13]. The cutoff value for ER positivity and PR positivity was at least 1% positive tumor cells with nuclear staining [14]. HER2 status was evaluated semi-quantitatively by IHC according to the ASCO/CAP guidelines [15–17]. Tumors with IHC HER2 2+ were further examined by fluorescence in situ hybridization (FISH), and HER2 positivity was defined as IHC HER2 3+ or FISH +. Ki-67 expression was scored as the percentage of positive invasive tumor cells with any nuclear staining and recorded as mean percentage of positive cells.

### Follow-up

All patients were followed up by outpatient visit or call every 3 months for the first 2 years after surgery, every 6 months between the 3rd and 5th years, then annually until death. Disease-free survival (DFS) was computed from the date of surgery to the date of the following events: locoregional recurrence, contralateral breast cancer, secondary non-breast malignancy, distant recurrence at any site, and death of any cause. Overall survival (OS) was defined as the time period from the date of surgery to the date of death of any cause. Diagnosis of recurrence or metastasis was generally based on patient’s radiographic images and/or pathological biopsies when accessible. Last follow-up was conducted in April 2021.

### Statistical analysis

Descriptive characteristics of categorical variables were tested using chi-square test or Fisher’s exact test. Multivariate logistic regression analysis was used to compare the baseline clinic-pathological features and therapies between these two groups. The estimated 5-year DFS and OS were calculated by Kaplan-Meier analysis. Cox proportional hazards regression analysis was performed to calculate independent prognostic factors in ER+/HER2+ patients treated with Trastuzumab. All statistical procedures were performed using SPSS software, version 22.0 (SPSS Company, Chicago, IL). *P* <  0.05 was considered statistically significant.

## Results

### Patients characteristics

**Fig. 1:**
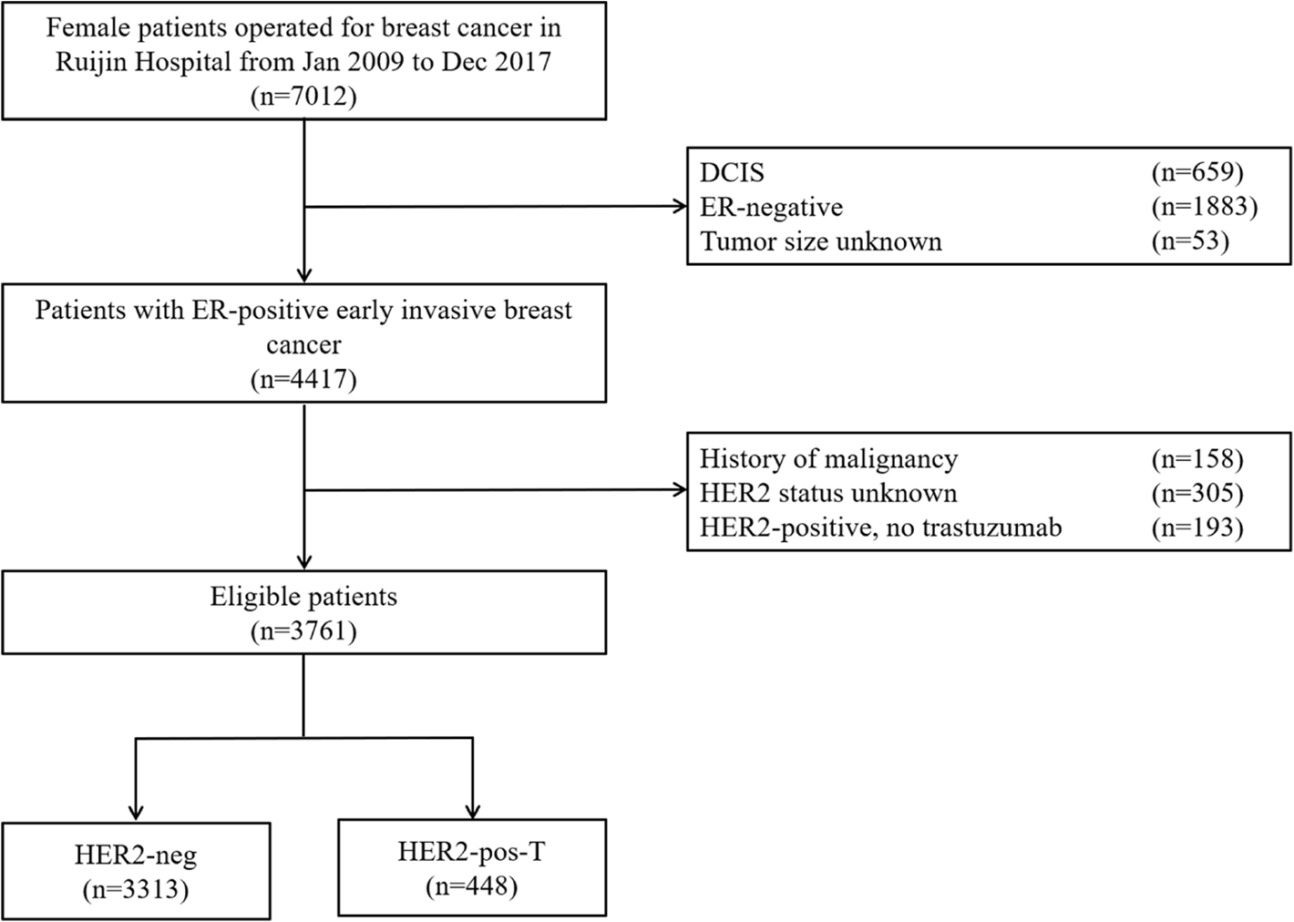
Flowchart of the 3761 patients included in the study. Abbreviations: DCIS, ductal carcinoma in situ; ER, estrogen receptor; HER2, human epidermal growth factor receptor 2.

Between January 2009 and December 2017, 7012 consecutive female breast cancer patients received surgery. A total of 3761 patients with early invasive breast cancer were included (Fig. 1). The study flowchart was shown in the Fig. 1. The median age was 55 (IQR 46–64) years, and 1562 patients (41.5%) were pre/peri-menopausal at diagnosis. Tumors > 2.0 cm were found in 2251 patients (59.5%), and 1346 patients (35.8%) had positive axillary lymph nodes (ALNs). There were 796 patients (21.2%) with grade III disease and 284 (7.6%) with lympho-vascular invasion (LVI). All patients had ER+ disease, of whom 113 (3.0%) had ER staining less than 10% breast cancer cells and 3154 (83.9%) had tumors expressing ER in more than 50% cells. PR less than 20% was found in 1671 (44.4%) patients and 2016 (53.6%) patients had Ki-67 ≥ 14%.

### Clinicopathological characteristics among ER+/HER2+ patients treated with trastuzumab

Among the eligible patients, 448 (11.9%) had ER+/HER2+ tumors and received trastuzumab treatment, while the remaining 3313 (88.1%) cases were ER+/HER2-. Age, menstrual status, tumor size, ALN status, histological grade, pathological type, LVI status, ER level, PR level, and Ki-67 level were significantly different between the HER2-neg and HER2-pos-T groups (all *P* <  0.01, Table 1). Multivariate logistic regression analysis showed that the overall distribution of menstrual status, histological grade, LVI status, ER level, PR level, and Ki-67 level had a significant difference between these two groups (Supplementary Table S1). Compared with the HER2-neg group, patients in the HER2-pos-T group were more likely to be pre/peri-menopausal (Odds ratio [OR] 0.68, 95% confidence interval [CI] 0.55–0.85, *P* <  0.001) and they tended to have higher histological grade (OR 1.41, 95% CI 1.11–1.79), LVI (OR 1.67, 95% CI 1.19–2.33, *P* <  0.001) and higher Ki-67 level (OR 4.37, 95% CI 3.28–5.82, *P* <  0.001). By contrast, ER (OR 0.37, 95% CI 0.24–0.57, *P* <  0.001) and PR (OR 0.42, 95% CI 0.33–0.53, *P* <  0.001) expression levels were lower in the HER2-pos-T group compared with the ER+/HER2- group.

**Table 1:** Tumor and patient characteristics stratified by different groups

Characteristics	Total *n* = 3761 (%)	HER2-neg *n* = 3313 (%)	HER2-pos-T *n* = 448 (%)	*P* value
Age (y/o)	55 (46–64)	56 (46–65)	51 (44–59)	< 0.001
Menstrual status				< 0.001
Pre/Peri-	1562 (41.5)	1333 (40.2)	229 (51.1)	
Post-	2199 (58.5)	1980 (59.8)	219 (48.9)	
Histology type				< 0.001
IDC	3085 (82.0)	2679 (80.9)	406 (90.6)	
Non-IDC	676 (18.0)	634 (19.1)	42 (9.4)	
Histological grade				< 0.001
I/II	2395 (63.7)	164 (65.4)	231 (51.6)	
III	796 (21.2)	614 (18.5)	182 (40.6)	
NA	570 (15.1)	535 (16.1)	38 (7.8)	
Tumor size				< 0.001
≤ 2.0 cm	2251 (59.5)	2039 (61.5)	212 (47.3)	
> 2.0 cm	1510 (40.1)	1274 (38.5)	236 (52.7)	
ALN status				0.002
Negative	2415 (64.2)	2157 (65.1)	258 (57.6)	
Positive	1346 (35.8)	1156 (34.9)	190 (42.4)	
LVI				< 0.001
No	3477 (92.4)	3091 (93.3)	386 (86.2)	
Yes	284 (7.6)	222 (6.7)	62 (13.8)	
ER				< 0.001
1–9%	113 (3.0)	66 (2.0)	47 (10.5)	
10–49%	494 (13.1)	389 (11.7)	105 (23.4)	
≥ 50%	3154 (83.9)	2858 (86.3)	296 (66.1)	
PR				< 0.001
< 20%	1671 (44.4)	1363 (41.1)	308 (68.8)	
≥ 20%	2090 (55.6)	1950 (58.9)	140 (31.3)	
Ki-67				< 0.001
< 14%	1745 (46.4)	1679 (50.7)	66 (14.7)	
≥ 14%	2016 (53.6)	1634 (49.3)	382 (85.3)	

HER2, human epidermal growth factor receptor 2; BCS, breast-conserving surgery; IDC, invasive ductal carcinoma; ALN, axillary lymph node; LVI, lymph-vascular invasion; ER, estrogen receptor; PR, progesterone receptor; y/o, years old

### Treatment among ER+/HER2+ patients treated with trastuzumab

Compared with the HER2-neg group, patients in the HER2-pos-T group were more likely to be treated with neo-adjuvant therapy (11.8% versus [vs.] 5.7%, *P* <  0.001) and mastectomy (77.9% vs. 66.9%, *P* <  0.001, Supplementary Table S2). Radiotherapy was applied for 51.2% and 59.6% patients in the HER2-neg and HER2-pos-T groups, respectively (*P* <  0.001). There were 91.3% patients in the HER2-pos-T group receiving chemotherapy, which was higher than the rate in the HER2-neg group (54.0%, *P* <  0.001). No statistically significant difference was observed in terms of adjuvant endocrine therapy between these two groups (93.8% vs. 93.5%, *P* = 0.814).

### Prognosis of ER+/HER2+ patients treated with trastuzumab

**Fig. 2:**
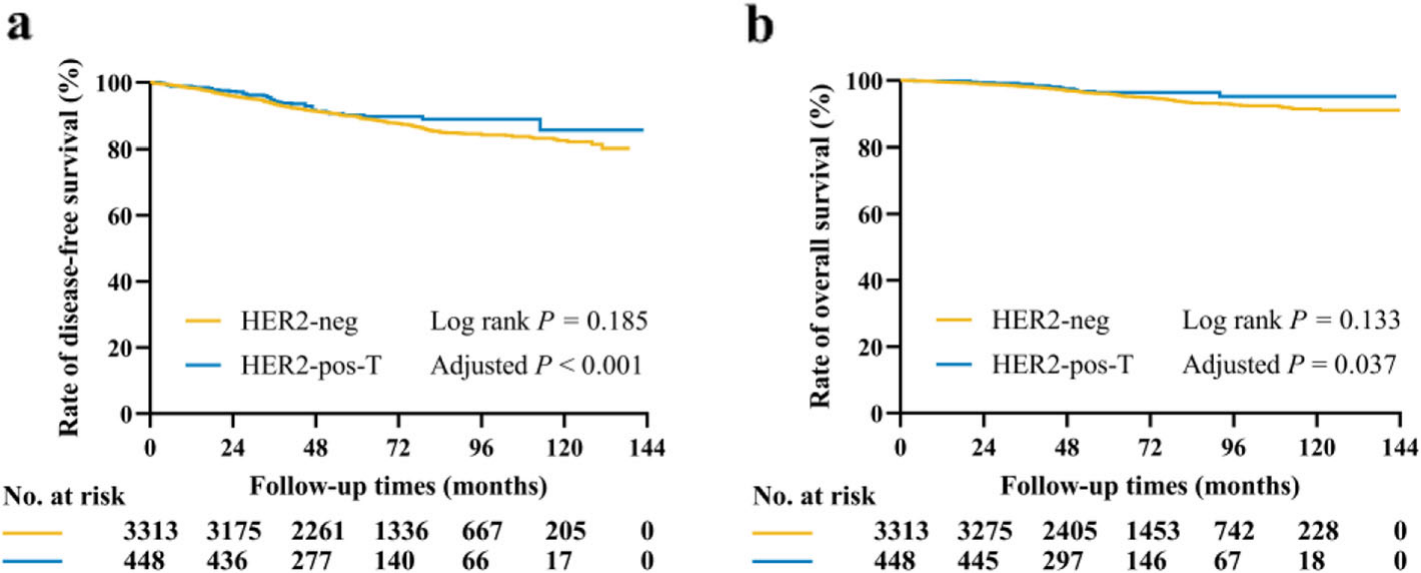
Kaplan-Meier estimates of DFS and OS in the whole population. **a**. The estimated 5-year DFS rate was 89.7% for HER2- patients and 90.2% for HER2+ patients treated with trastuzumab (*P* = 0.185). HER2-pos-T was associated with significantly better DFS (*P* < 0.001) after adjusting neo-adjuvant therapy, breast surgery, tumor size, ALN status, histological grade, LVI, ER level, PR level, Ki-67 level, radiotherapy, chemotherapy, and endocrine therapy. **b**. The estimated 5-year OS rate was 96.0% in the HER2-neg group and 96.3% in the HER2-pos-T group (*P* = 0.133). HER2-pos-T was associated with significantly better OS (*P* = 0.037) after adjusting menstrual status, neo-adjuvant therapy, breast surgery, tumor size, ALN status, histological grade, LVI, ER level, PR level, Ki-67 level, radiotherapy, and endocrine therapy

After a median follow-up of 62 (IQR 43–87) months, 443 DFS events were recorded (Table 2). The estimated 5-year DFS rate was 89.7% for HER2- patients and 90.2% for HER2+ patients treated with trastuzumab (*P* = 0.185, Fig. 2a). Univariate analysis found that neo-adjuvant therapy, breast surgery, tumor size, ALN status, histological grade, LVI, ER level, PR level, Ki-67 level, radiotherapy, chemotherapy, and endocrine therapy were associated with DFS (all *P* <  0.05, Supplementary Table S3). Neo-adjuvant therapy (HR 2.51, 95% CI: 1.91–3.30, *P* <  0.001), tumor > 2.0 cm (HR 1.54, 95% CI: 1.26–1.88, *P* <  0.001), positive ALN (HR 1.58, 95% CI: 1.29–1.93, *P* <  0.001), grade III (HR 1.44, 95% CI: 1.15–1.81), and Ki-67 ≥ 14% (HR 1.49, 95% CI: 1.21–1.85, *P* <  0.001) were associated with worse DFS in multivariable analysis, while PR ≥ 20% (HR 0.82, 95% CI: 0.67–1.00, *P* = 0.050) and endocrine therapy (HR 0.52, 95% CI: 0.37–0.73, *P* <  0.001) were associated with significantly better DFS (Table 3). More importantly, patients with HER2+ disease treated with trastuzumab had a significantly better DFS than patients in the ER+/HER2- group (HR 0.52, 95% CI: 0.37–0.73, *P* <  0.001, Table 3).

**Table 2:** DFS and OS events stratified by different groups

	Overall *n* = 3761 (%)	HER2-neg *n* = 3313 (%)	HER2-pos-T *n=* 448 (%)
**DFS events**			
No recurrence	3318 (88.2)	2911 (87.9)	407 (90.8)
Local-regional recurrence	73 (1.9)	63 (1.9)	10 (2.2)
Contralateral breast cancer	38 (1.0)	36 (1.0)	2 (0.5)
Second non-breast malignancy	56 (1.5)	52 (1.6)	4 (1.0)
Distant recurrence	198 (5.3)	179 (5.4)	19 (4.2)
Death without recurrence	78 (2.1)	72 (2.2)	6 (1.3)
**OS events**			
Alive	3570 (94.9)	3136 (94.7)	434 (96.9)
Death of any cause	191 (5.1)	177 (5.3)	14 (3.1)
Death with recurrence	123 (3.0)	105 (3.1)	8 (1.8)
Death without recurrence	78 (2.1)	72 (2.2)	6 (1.3)

HER2, human epidermal growth factor receptor 2; DFS, disease-free survival; OS, overall survival

**Table 3:** Multivariate analysis of factors associated with DFS and OS

Characteristics	DFS		OS	
	HR (95% CI)	*P* value	HR (95% CI)	*P* value
Menstrual status (Post - vs. Pre/Peri-)	/	/	2.33 (1.69–3.22)	< 0.001
Neo-adjuvant therapy (Yes vs. No)	2.51 (1.91–3.30)	< 0.001	2.39 (1.61–3.56)	< 0.001
Breast surgery (BCS vs. Mastectomy)	1.00 (0.77–1.30)	0.992	0.73 (0.47–1.12)	0.149
Tumor size (> 2.0 cm vs. ≤ 2.0 cm)	1.54 (1.26–1.88)	< 0.001	1.81 (1.32–2.48)	< 0.001
ALN status (Positive vs. Negative)	1.58 (1.29–1.93)	< 0.001	2.15 (1.57–2.93)	< 0.001
Histological grade		< 0.001		< 0.001
III vs. I/II	1.44 (1.15–1.81)		1.69 (1.19–2.40)	
NA vs. I/II	1.58 (1.21–2.06)		2.02 (1.36–3.01)	
LVI (Yes vs. No)	1.26 (0.90–1.75)	0.176	1.55 (0.94–2.54)	0.084
ER		0.074		< 0.001
10–49% vs. 1–9%	1.18 (0.72–1.94)		0.95 (0.52–1.73)	
≥ 50% vs. 1–9%	0.89 (0.55–1.45)		0.44 (0.24–0.78)	
PR (≥ 20% vs. < 20%)	0.82 (0.67–1.00)	0.050	0.77 (0.55–1.07)	0.118
Ki-67 (≥ 14% vs. < 14%)	1.49 (1.21–1.85)	< 0.001	1.43 (1.02–2.00)	0.037
Radiotherapy (Yes vs. No)	0.97 (0.78–1.21)	0.790	1.09 (0.74–1.59)	0.675
Chemotherapy (Yes vs. No)	1.15 (0.91–1.46)	0.236	0.94 (0.66–1.35)	0.744
Endocrine therapy (Yes vs. No)	0.56 (0.41–0.76)	< 0.001	0.46 (0.30–0.71)	< 0.001
Group (HER2-pos-T vs. HER2-neg)	0.52 (0.37–0.73)	< 0.001	0.38 (0.22–0.67)	0.037

HER2, human epidermal growth factor receptor 2; BCS, breast-conserving surgery; ALN, axillary lymph node; LVI, lymph-vascular invasion; ER, estrogen receptor; PR, progesterone receptor; y/o, years old

A total of 191 deaths were recorded (177 in the HER2-neg group and 14 in the HER2-pos-T group, Table 2). Among these patients, 72 (40.7%) in the HER2-neg group and 6 (42.9%) in the HER2-pos-T group didn’t have tumor recurrence. There was no significant OS difference between these two groups. The estimated 5-year OS rate was 96.0% in the HER2-neg group and 96.3% in the HER2-pos-T group (*P* = 0.133, Fig. 2b). Univariate analysis identified that menstrual status, neo-adjuvant therapy, breast surgery, tumor size, ALN status, histological grade, LVI, ER level, PR level, Ki-67 level, radiotherapy, and endocrine therapy were associated with OS (all *P* <  0.05, Supplementary Table S3). Multivariable analysis showed that post-menopause (HR 2.33, 95% CI: 1.69–3.22, *P* <  0.001), neo-adjuvant therapy (HR 2.39, 95% CI: 1.61–3.56, *P* <  0.001), tumor > 2.0 cm (HR 1.81, 95% CI: 1.32–2.48, *P* <  0.001), positive ALNs (HR 2.15, 95% CI: 1.97–2.93, *P* <  0.001), grade III (HR 1.69, 95% CI: 1.19–2.40), and Ki-67 ≥ 14% (HR 1.43, 95% CI: 1.02–2.00, *P* = 0.037) were associated with worse OS, while ER ≥ 50% (HR 0.44, 95% CI: 0.24–0.78) and endocrine therapy (HR 0.46, 95% CI: 0.30–0.71, *P* <  0.001) were associated with significantly better OS (Table 3). Similarly, patients in the HER2-pos-T group had significantly better OS than those in the HER2-neg group (HR 0.38, 95% CI: 0.22–0.67, *P* = 0.037, Table 3).

### Prognostic effect of HER2 status in ER+ patients according to different subtypes

Prognostic effect interactions were not significantly different between HER2 status and certain subgroups including age, menstrual status, tumor size, ALN status, tumor grade, ER level, PR level, and Ki-67 level (Supplementary Fig. S1).

**Fig. 3:**
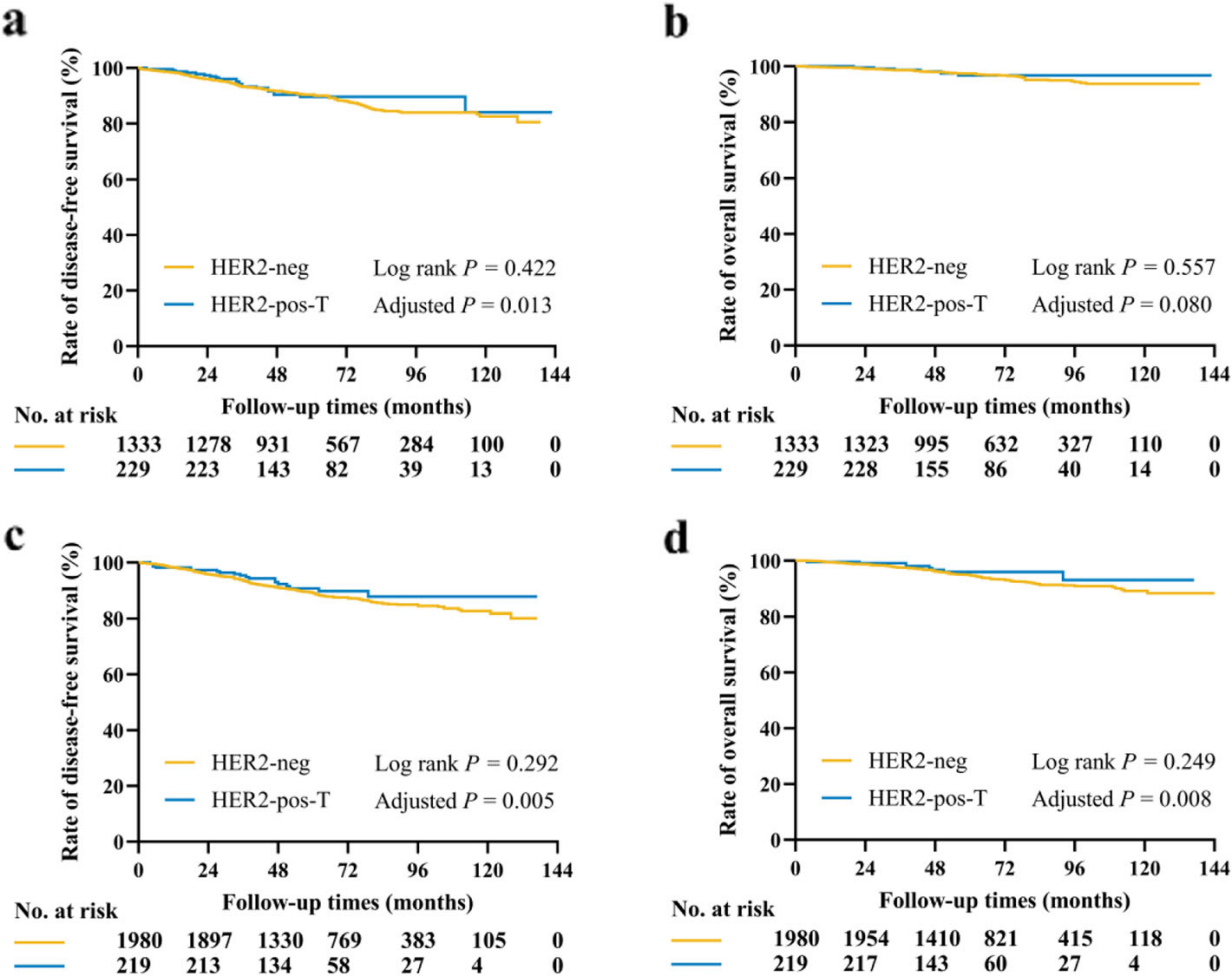
Kaplan-Meier estimates of DFS and OS for pre/peri-menopausal patients (**a** and **b**) and post-menopausal patients (c and d). **a**. The estimated 5-year DFS rate was 90.3% for HER2- patients and 89.6% for HER2+ patients treated with trastuzumab (*P* = 0.422). HER2-pos-T was associated with significantly better DFS in multivariate analysis (*P* = 0.013). **b**. The estimated 5-year OS rate was 97.4% in the HER2-neg group and 96.7% in the HER2-pos-T group (*P* = 0.557). No difference was observed between these two groups in multivariate analysis (*P* = 0.080). **c**. The estimated 5-year DFS rate was 89.3% for HER2- patients and 90.7% for HER2+ patients treated with trastuzumab (*P* = 0.292). HER2-pos-T was associated with significantly better DFS in multivariate analysis (*P* = 0.005). **d**. The estimated 5-year OS rate was 95.0% in the HER2-neg group and 96.0% in the HER2-pos-T group (*P* = 0.249). HER2-pos-T was associated with significantly better OS in multivariate analysis (*P* = 0.008)

For pre/peri-menopausal patients, the estimated 5-year DFS rate was 90.3% in the HER2-neg group and 89.6% in HER2-pos-T group (*P* = 0.422, Fig. 3a). In univariate analysis, neo-adjuvant therapy, breast surgery, tumor size, ALN status, histological grade, LVI, ER level, PR level, Ki-67 level, radiotherapy, chemotherapy, and endocrine therapy were prognostic for DFS (Supplementary Table S4). As shown in the Table 4, neo-adjuvant therapy, tumor > 2.0 cm, LVI were unfavorable prognostic factor in multivariate analysis, while endocrine therapy (HR 0.41, 95% CI: 0.26–0.62, *P* <  0.001) and HER2-pos-T (HR 0.56, 95% CI: 0.36–0.89, *P* = 0.013) were associated with significantly better DFS. At 5 year, 97.4% patients in the HER2-neg group and 96.7% in the HER2-pos-T group were alive (*P* = 0.577, Fig. 3b). Neo-adjuvant therapy, breast surgery, tumor size, ALN status, histological grade, LVI, ER level, PR level, and endocrine therapy affected OS in univariate analysis (Supplementary Table S4). Neo-adjuvant therapy, breast surgery, tumor size, ALN status, histological grade, and endocrine therapy remained independent prognostic factors in multivariate analysis (Table 4). After adjusting these factors, there was no obvious difference in terms of OS between these two groups (HR 0.46, 95% CI: 0.19–1.16, *P* = 0.080).

**Table 4:** Multivariate analysis of factors associated with DFS and OS in pre/peri- menopausal patients

Characteristics	DFS		OS	
	HR (95% CI)	*P* value	HR (95% CI)	*P* value
Neo-adjuvant therapy (Yes vs. No)	3.74 (2.56–5.46)	< 0.001	2.27 (1.22–4.21)	0.009
Breast surgery (BCS vs. Mastectomy)	0.84 (0.59–1.21)	0.841	0.42 (0.17–1.00)	0.050
Tumor size (> 2.0 cm vs. ≤ 2.0 cm)	1.84 (1.34–2.51)	< 0.001	2.89 (1.46–5.71)	0.002
ALN status (Positive vs. Negative)	1.14 (0.78–1.65)	0.503	2.10 (1.16–3.83)	0.015
Histological grade		0.328		0.013
III vs. I/II	1.30 (0.92–1.84)		2.45 (1.34–4.50)	
NA vs. I/II	1.06 (0.65–1.71)		1.92 (0.87–4.27)	
LVI (Yes vs. No)	1.76 (1.16–2.67)	0.008	1.82 (0.87–3.82)	0.112
ER		0.146		0.055
10–49% vs. 1–9%	0.96 (0.47–1.93)		0.65 (0.25–1.73)	
≥ 50% vs. 1–9%	0.70 (0.35–1.39)		0.37 (0.14–0.98)	
PR (≥ 20% vs. < 20%)	0.86 (0.62–1.20)	0.384	0.83 (0.44–1.55)	0.559
Ki-67 (≥ 14% vs. < 14%)	1.51 (1.09–2.09)	0.013	/	/
Radiotherapy (Yes vs. No)	1.23 (0.86–1.77)	0.254	/	/
Chemotherapy (Yes vs. No)	1.50 (1.04–2.17)	0.032	/	/
Endocrine therapy (Yes vs. No)	0.41 (0.26–0.62)	< 0.001	0.37 (0.19–0.73)	0.004
Group (HER2-pos-T vs. HER2-neg)	0.56 (0.36–0.89)	0.013	0.46 (0.19–1.10)	0.080

HER2, human epidermal growth factor receptor 2; BCS, breast-conserving surgery; ALN, axillary lymph node; LVI, lymph-vascular invasion; ER, estrogen receptor; PR, progesterone receptor; y/o, years old

Regarding post-menopausal patients, the estimated 5-year DFS rate was 89.3% and 90.7% in the HER2-neg group and HER2-pos-T group, respectively (*P* = 0.292, Fig. 3c). Univariate analysis showed that neo-adjuvant therapy, tumor size, ALN status, histological grade, ER level, PR level, Ki-67 level, radiotherapy, chemotherapy, and endocrine therapy were associated with DFS (Supplementary Table S4). As shown in Table 5, multivariate analysis suggested that neo-adjuvant therapy, tumor size, ALN status, histological grade, ER level, Ki-67 level, and endocrine therapy were independent prognostic factors for DFS. Notably, patients in the HER2-pos-T group had a significantly better DFS (HR 0.52, 95% CI: 0.32–0.85, *P* = 0.005). At 5 year, there were 95.0% and 96.0% patients who were free from death in the two groups, respectively (*P* = 0.249, Fig. 3d). Neo-adjuvant therapy, breast surgery, tumor size, ALN status, histological grade, ER level, PR level, Ki-67 level, radiotherapy, and endocrine therapy were associated with OS in univariate analysis (Supplementary Table S4). In multivariate analysis, neo-adjuvant therapy, tumor size, ALN status, histological grade, ER level, and endocrine therapy remained as independent prognostic factors (Table 5). Similarly, patients with HER2+ disease and treated with trastuzumab had significantly better OS compared with patients in the HER2-neg group (HR 0.37, 95% CI: 0.17–0.77, *P* = 0.008).

**Table 5:** Multivariate analysis of factors associated with DFS and OS in post- menopausal patients

Characteristics	DFS		OS	
	HR (95% CI)	*P* value	HR (95% CI)	*P* value
Neo-adjuvant therapy (Yes vs. No)	2.24 (1.51–3.30)	< 0.001	2.18 (1.30–3.66)	0.003
Breast surgery (BCS vs. Mastectomy)	/	/	0.92 (0.59–1.44)	0.722
Tumor size (> 2.0 cm vs. ≤ 2.0 cm)	1.46 (1.13–1.89)	< 0.001	1.53 (1.06–2.20)	0.024
ALN status (Positive vs. Negative)	1.74 (1.34–2.26)	< 0.001	2.23 (1.55–3.19)	< 0.001
Histological grade		< 0.001		0.004
III vs. I/II	1.54 (1.13–2.09)		1.53 (1.00–2.36)	
NA vs. I/II	2.05 (1.48–2.85)		2.08 (1.31–3.29)	
ER		0.043		< 0.001
10–49% vs. 1–9%	1.56 (0.76–3.21)		1.01 (0.47–2.18)	
≥ 50% vs. 1–9%	1.02 (0.51–2.02)		0.45 (0.22–0.93)	
PR (≥ 20% vs. < 20%)	0.84 (0.64–1.09)	0.194	0.76 (0.52–1.13)	0.177
Ki-67 (≥ 14% vs. < 14%)	1.50 (1.14–1.99)	0.004	1.39 (0.94–2.07)	0.098
Radiotherapy (Yes vs. No)	0.91 (0.66–1.25)	0.549	1.13 (0.71–1.79)	0.605
Chemotherapy (Yes vs. No)	1.12 (0.83–1.52)	0.466	/	/
Endocrine therapy (Yes vs. No)	0.56 (0.37–0.86)	0.007	0.53 (0.31–0.91)	0.021
Group (HER2-pos-T vs. HER2-neg)	0.52 (0.32–0.85)	0.005	0.37 (0.17–0.77)	0.008

HER2, human epidermal growth factor receptor 2; BCS, breast-conserving surgery; ALN, axillary lymph node; ER, estrogen receptor; PR, progesterone receptor; y/o, years old

Among 243 patients who received neo-adjuvant therapy, the 5-year DFS rate was 71.9% for those in the HER2-pos-T group and 71.3% for those in the HER2-neg group (*P* = 0.463, Supplementary Fig. S2a). In multivariate analysis, patients in the HER2-pos-T group had significantly superior DFS (HR 0.51, 95% CI: 0.27–0.97, *P* = 0.041). The 5-year OS rate was 87.7% and 85.1% for the two arms, respectively (*P* = 0.341, Supplementary Fig. S2b). There was no difference between the two groups in OS (HR 0.40, 95% CI: 0.15–1.09, *P* = 0.073).

The 5-year DFS rate was 91.9% for ER+/HER2+ patients treated with chemotherapy and trastuzumab and 87.6% for ER+/HER2- patients who received chemotherapy (*P* = 0.011, Supplementary Fig. S2c). The 5-year OS rate was 97.3% and 95.2% for the two arms, respectively (*P* = 0.002, Supplementary Fig. S2d). Patients in the HER2-pos-T group had significantly superior DFS (HR 0.51, 95% CI: 0.35–0.74, *P* <  0.001) and OS (HR 0.32, 95% CI: 0.17–0.63, *P* = 0.001) than those in the HER2-neg group among the chemotherapy subset.

For patients receiving radiotherapy, the 5-year DFS rate was 89.8% for the HER2-pos-T group and 87.9% for the HER2-neg group (*P* = 0.216, Supplementary Fig. S2e). The 5-year OS rate was 96.6% and 95.4% for the two arms, respectively (*P* = 0.127, Supplementary Fig. S2f). Similarly, DFS (HR 0.53, 95% CI: 0.35–0.81, *P* = 0.003) and OS (HR 0.32, 95% CI: 0.15–0.68, *P* = 0.003) were significantly better in favor of the HER2-pos-T group.

The 5-year estimates of DFS rates were 89.7% for the HER2-pos-T group and 91.5% for the HER2-neg group among those treated with a selective estrogen receptor modulator (SERM) (*P* = 0.573, Supplementary Fig. S3a). Estimated 5-year OS rates were 96.8% and 97.7% for the two arms, respectively (*P* = 0.651, Supplementary Fig. S3b). Patients in the HER2-pos-T group had significantly better DFS (HR 0.52, 95% CI: 0.28–0.96, *P* = 0.038) while OS (HR 0.46, 95% CI: 0.14–1.58, *P* = 0.219) was comparable between the two arms. For the 297 patients assigned SERM-AI, the 5-year DFS, OS rates were 95.2%, 97.2% for the HER2-pos-T group and 95.0%, 99.1% for the HER2-neg group, respectively (*P* = 0.605, 0.228, Supplementary Fig. S3c, d). No differences were observed in DFS (HR 0.47, 95% CI: 0.09–2.44, *P* = 0.371) and OS (HR 3.03, 95% CI: 0.36–25.15, *P* = 0.305) in multivariate analysis. Among patients receiving an AI, the DFS rate was 90.0% and 89.1% for the HER2-pos-T and HER2-neg groups (*P* = 0.334, Supplementary Fig. S3e). A total of 97.7% in the HER2-pos-T group and 95.3% in the HER2-neg group were alive at 5 years (*P* = 0.042, Supplementary Fig. S3f). DFS (HR 0.58, 95% CI: 0.37–0.93, *P* = 0.023) and OS (HR 0.26, 95% CI: 0.11–0.65, *P* = 0.004) of the HER2-pos-T group were superior to that of the HER2-neg group.

### Predictive effect of HER2 status in ER+ patients

There were 256 pre-menopausal women in the present cohort who were treated with OFS (75 with SERM, 16 with SERM-AI, and 165 with AI), and 1211 received endocrine therapy alone (Supplementary Table S2). The rate of OFS utilization was higher in the HER2-pos-T group than that in the HER2-neg group (23.3% vs. 16.5%, *P* = 0.016). For HER2- patients, 86.5% in the OFS arm and 92.2% in the Non-OFS arm were disease-free at 5 year (*P* = 0.013, Supplementary Fig. S4a). While the 5-year DFS rate was 89.5% in the OFS arm and 90.2% in the Non-OFS arm among HER2+ patients (*P* = 0.995, Supplementary Fig. S4b). There was no interaction between HER2 status and OFS benefit in terms of DFS (*P* = 0.556). For HER2- patients, 97.8% in the OFS arm and 98.0% in the Non-OFS arm were alive at 5 year (*P* = 0.245, Supplementary Fig. S4c). The rates among HER2+ patients were 100.0% and 96.3%, respectively (*P* = 0.277, Supplementary Fig. S4d). Similarly, no interaction between HER2 status and OFS benefit in OS were observed (*P* = 0.965).

## Discussion

Patients with ER+/HER2+ breast cancer had a worse prognosis compared to those with ER+/HER2- disease in the pre-trastuzumab treatment era, who were more frequently treated with chemotherapy and escalation endocrine therapy. Consistent with previous studies, our current study found that ER+/HER2+ tumors were associated with pre/peri-menopause, higher histological grade, LVI, higher Ki-67 level, lower ER and PR levels. After adjusting for these characteristics, the DFS and OS rates were significantly better for ER+/HER2+ patients treated with trastuzumab than those with ER+/HER2- tumor, indicating HER2-positivity itself may not be an adverse factor for ER+ patients if they were treated with trastuzumab, which may guide further clinical treatment decision making.

Previous reports validated that ER+/HER2+ breast cancers were associated with more unfavorable clinical features compared with ER+/HER2- diseases. A study based on the SEER database from 2010 to 2014 revealed that age < 35 y/o (3.6% vs. 1.3%), tumor > 2.0 cm (48.8% vs. 36.8%), stage III/IV (21.5% vs. 13.7%), tumor grade III (47.9% vs. 17.4%), and less PR expression (72.9% vs. 87.7%) was more common in the HR+ /HER2+ subgroup compared to HR+ /HER2– patients [18]. Likewise, a meta-analysis showed that the co-expression of HER2 and HR was associated with age < 40 y/o (12% vs. 6%), tumor > 2.0 cm (53% vs. 39%), positive ALN (56% vs. 42%), and tumor grade III (56% vs. 29%) compared to HR+ /HER2– patients [19]. Our results showed that ER+/HER2+ patients were significantly associated with pre/peri-menopause, higher histological grade, LVI, and higher Ki-67 level compared with ER+/HER2- patients. Moreover, ER+/HER2+ patients had lower ER and PR expression levels in our cohort, indicating ER+/HER2+ patients had unfavorable prognostic factors.

To the best of our knowledge, this is the first study comparing the prognosis of ER+/HER2+ patients treated with trastuzumab and those patients with ER+/HER2- tumors. Previously, two trials that assessed the efficacy of trastuzumab prospectively included a parallel observational cohort in which women with HER2- diseases received the same chemotherapy as did the HER2+ group. The 3-year results of the FinHer study indicated that survival free of distant disease was comparable among women with HER2+ cancer who received trastuzumab and those with HER2- cancer (HR 1.09, 95% CI 0.52–2.29, *P* = 0.82) [20]. In the NOAH trial, the estimated 5-year rate of event-free survival was 58% in the HER2+ treated with trastuzumab group and 61% in the HER2- group [21]. However, because of the differences in clinic-pathological factors between HER2+ patients and those with HER2- tumors, the actual efficacy of trastuzumab on prognosis remained unknown. In the present study, ER+/HER2+ patients treated with trastuzumab had significantly better 5-year DFS (HR 0.52, 95% CI: 0.37–0.73, *P* <  0.001) and OS (HR 0.38, 95% CI: 0.22–0.67, *P* = 0.037) rates than those with ER+/HER2- tumor in multivariable analysis by adjusting clinicopathological factors, indicating HER2 positivity was no longer an adverse factor for ER+ patients if they were treated with trastuzumab, which may guide further clinical treatment decision making.

For pre-menopausal patients with high risk factors, adding OFS with endocrine therapy was recommended according to the SOFT and TEXT trials [22–27]. Several clinical trials showed heterogeneity of OFS treatment benefit according to HER2 status [23–27]. Data from the SOFT trial suggested that HER2+ patients received a greater DFS benefit from the addition of OFS to tamoxifen compared with HER2- patients (HR 0.41 vs. 0.83, interaction *P* = 0.04) [26]. However, the joint analysis of SOFT and TEXT showed that more treatment benefit with exemestane plus OFS than tamoxifen plus OFS was found in patients with HER2- cancers (interaction *P* = 0.014) [26]. Similarly, a statistically significant treatment benefit difference according to the HER2 status was found in the HOBOE trial and OFS plus letrozole was more effective compared with OFS plus tamoxifen in the HER2- patients [25]. In the SOFT and TEXT trials, 60.1% ER+/HER2+ patients received anti-HER2 therapy. In our study, all ER+/HER2+ patients were treated with trastuzumab and they had significantly better disease outcome compared with HER2- patients, suggesting that only HER2 positivity itself may not be enough to recommend additional OFS treatment in pre-menopausal ER+/HER2+ patients if they were treated with trastuzumab.

In the present study, we observed a higher utilization rate of OFS among ER+/HER2+ patients treated with trastuzumab than ER+/HER2- patients. However, HER2 status seemed to provide no predictive information for OFS benefit with the presence of trastuzumab treatment. These results also suggested that HER2 positivity itself might not necessarily be an indicator for escalation endocrine therapy, which needed further research.

There were several potential limitations in this study. First, the study was retrospective and single-centered, which may lead to unavoidable bias in basic characteristics and treatment patterns between different arms. For example, 99.6% of the patients in the HER2-pos-T group received chemotherapy, whereas the percentage of chemotherapy was 55.6% in the HER2-neg group. Secondly, 136 ER+/HER2+ patients who didn’t receive trastuzumab (small tumor or patients reject to receive treatment) were excluded from the analysis. Moreover, the follow-up was still too short to clarify the effect of trastuzumab on disease outcomes, especially for ER+ disease, which needed additional follow-up.

## Conclusions

Compared with ER+/HER2- breast cancers, ER+/HER2+ tumors were associated with unfavorable clinic-pathological factors. However, disease outcome of ER+/HER2+ patients treated with trastuzumab was better than those in the ER+/HER2- group, which was consistent in the pre- or post-menopausal women, indicating escalation of endocrine therapy may not be necessary for those ER+/HER2+ patients treated with trastuzumab, which warrants further clinical evaluation.

## Supplementary Information

**Additional file 1.**

## Data Availability

The datasets used and/or analysed during the current study are available from the corresponding author on reasonable request.
