# Supplementary material for: Clinical characteristics and disease outcomes in ER+ breast cancer: a comparison between HER2+ patients treated with trastuzumab and HER2- patients
Source: BMC Cancer. 2021 Jul 13;21:807. doi: 10.1186/s12885-021-08555-4 (PMC8278709; doi:10.1186/s12885-021-08555-4)
Supplement: Supplementary file 1 — Additional file 1. [file 12885_2021_8555_MOESM1_ESM.doc]

**Supplementary Materials**

**Clinical characteristics and disease outcomes in ER+ breast cancer: a comparison between HER2+ patients treated with trastuzumab and HER2- patients**

Shuai Li ^a^, Jiayi Wu ^a^, Ou Huang ^a^, Jianrong He ^a^, Li Zhu ^a^, Weiguo Chen ^a^, Yafen Li ^a^, Xiaosong Chen ^a^,*, Kunwei Shen ^a^,**

**Supplementary Table S1**

Multivariate logistic regression analysis of tumor characteristics in the HER2-pos-T group compared with the HER2-neg group.

| Characteristics | OR (95% CI) | *P* value |
| --- | --- | --- |
| Menstrual status (Post - *vs.* Pre/Peri-) | 0.68 (0.55-0.85) | 0.001 |
| Histology type (Non-IDC *vs.* IDC) | 0.75 (0.44-1.27) | 0.280 |
| Tumor size (> 2.0 cm *vs.* ≤ 2.0 cm) | 1.22 (0.97-1.52) | 0.085 |
| ALN status (Positive *vs.* Negative) | 0.88 (0.70-1.10) | 0.266 |
| Histological grade |  | < 0.001 |
| Ⅲ *vs.* Ⅰ/Ⅱ | 1.41 (1.11-1.79) |  |
| NA *vs.* Ⅰ/Ⅱ | 0.92 (0.52-1.64) |  |
| LVI (Yes *vs.* No) | 1.67 (1.19-2.33) | 0.003 |
| ER |  | < 0.001 |
| 10-49% *vs.* 1-9% | 0.69 (0.43-1.09) |  |
| ≥ 50% *vs.* 1-9% | 0.37 (0.24-0.57) |  |
| PR (≥ 20% *vs.* < 20%) | 0.42 (0.33-0.53) | < 0.001 |
| Ki-67 (≥ 14% *vs.* < 14%) | 4.37 (3.28-5.82) | < 0.001 |

IDC, invasive ductal carcinoma; ALN: axillary lymph node; LVI, lymph-vascular invasion; ER, estrogen receptor; PR, progesterone receptor; OR, odds ratio; y/o, years old.

**Supplementary Table S2**

Treatment choices between the HER2-pos-T and HER2-neg breast cancer groups

| Characteristics | Total  n=3761 (%) | HER2-neg  n=3313 (%) | HER2-pos-T  n=448 (%) | *P* value |
| --- | --- | --- | --- | --- |
| Neoadjuvant therapy |  |  |  | < 0.001 |
| No | 3518 (93.5) | 3123 (94.3) | 395 (88.2) |  |
| Yes | 243 (6.5) | 190 (5.7) | 53 (11.8) |  |
| Breast surgery |  |  |  | < 0.001 |
| Mastectomy | 2565 (68.2) | 2216 (66.9) | 349 (77.9) |  |
| BCS | 1196 (31.8) | 1097 (33.1) | 99 (22.1) |  |
| Radiotherapy |  |  |  | 0.001 |
| No | 1794 (47.8) | 1613 (48.8) | 181 (40.4) |  |
| Yes | 1962 (52.2) | 1695 (51.2) | 267 (59.6) |  |
| Chemotherapy |  |  |  | < 0.001 |
| No | 1562 (41.6) | 1523 (46.0) | 39 (8.7) |  |
| Yes | 2194 (58.4) | 1785 (54.0) | 409 (91.3) |  |
| Endocrine therapy |  |  |  | 0.814 |
| No | 234 (6.2) | 205 (6.2) | 29 (6.5) |  |
| Yes | 3527 (93.8) | 3108 (93.8) | 419 (93.5) |  |
| Endocrine therapy regimen |  |  |  | 0.007 |
| SERM | 1147 (32.5) | 1016 (32.7) | 131 (31.3) |  |
| SERM-AI | 297 (8.4) | 245 (7.9) | 52 (12.4) |  |
| AI | 2083 (59.1) | 1847 (59.4) | 236 (56.3) |  |
| OFS |  |  |  | 0.016 |
| No | 1209 (82.5) | 1040 (83.5) | 169 (76.8) |  |
| Yes | 256 (17.5) | 205 (16.5) | 51 (23.2) |  |

HER2, human epidermal growth factor receptor 2; SERM, selective estrogen receptor modulator; AI, aromatase inhibitor; OFS, ovarian function suppression.

**Supplementary Table S3**

Univariate analysis of factors associated with DFS and OS in breast cancer patients.

| Characteristics | *P* value for DFS | *P* value for OS |
| --- | --- | --- |
| Menstrual status (Pre/Peri- *vs.* Post-) | 0.737 | < 0.001 |
| Neo-adjuvant therapy (No *vs.* Yes) | < 0.001 | < 0.001 |
| Breast surgery (Mastectomy *vs.* BCS) | 0.003 | < 0.001 |
| Histology type (IDC *vs.* Non-IDC) | 0.835 | 0.294 |
| Tumor size (≤ 2.0 cm *vs.* > 2.0 cm) | < 0.001 | < 0.001 |
| ALN status (Negative *vs.* Positive) | < 0.001 | < 0.001 |
| Histological grade (Ⅰ/Ⅱ *vs.* Ⅲ *vs.* NA) | < 0.001 | < 0.001 |
| LVI (No *vs.* Yes) | 0.001 | 0.001 |
| ER (1-9% *vs.* 10-49% *vs.* ≥ 50%) | < 0.001 | < 0.001 |
| PR (< 20% *vs.* ≥ 20%) | < 0.001 | < 0.001 |
| Ki-67 (< 14% *vs.* ≥ 14%) | < 0.001 | 0.010 |
| Radiotherapy (No *vs.* Yes) | 0.004 | 0.039 |
| Chemotherapy (No *vs.* Yes) | 0.001 | 0.131 |
| Endocrine therapy (No *vs.* Yes) | < 0.001 | < 0.001 |
| Group (HER2-neg *vs.* HER2-pos-T) | 0.185 | 0.131 |

HER2, human epidermal growth factor receptor 2; BCS, breast-conserving surgery; IDC, invasive ductal carcinoma; ALN, axillary lymph node; LVI, lymph-vascular invasion; ER, estrogen receptor; PR, progesterone receptor; y/o, years old.

**Supplementary Table S4**

Univariate analysis of factors associated with DFS and OS in pre/peri-menopausal and post-menopausal breast cancer patients.

| Characteristics | Pre/Peri- | |  | Post- | |
| --- | --- | --- | --- | --- | --- |
|  | DFS | OS |  | DFS | OS |
| Neo-adjuvant therapy (No *vs.* Yes) | < 0.001 | < 0.001 |  | < 0.001 | < 0.001 |
| Breast surgery (Mastectomy *vs.* BCS) | 0.004 | 0.001 |  | 0.152 | 0.020 |
| Histology type (IDC *vs.* Non-IDC) | 0.055 | 0.428 |  | 0.069 | 0.105 |
| Tumor size (≤ 2.0 cm *vs.* > 2.0 cm) | < 0.001 | < 0.001 |  | < 0.001 | < 0.001 |
| ALN status (Negative *vs.* Positive) | < 0.001 | < 0.001 |  | < 0.001 | < 0.001 |
| Histological grade (Ⅰ/Ⅱ *vs.* Ⅲ *vs.* NA) | < 0.001 | < 0.001 |  | < 0.001 | < 0.001 |
| LVI (No *vs.* Yes) | < 0.001 | < 0.001 |  | 0.552 | 0.118 |
| ER (1-9% *vs.* 10-49% *vs.* ≥ 50%) | 0.001 | < 0.001 |  | 0.006 | < 0.001 |
| PR (< 20% *vs.* ≥ 20%) | < 0.001 | < 0.001 |  | 0.003 | < 0.001 |
| Ki-67 (< 14% *vs.* ≥ 14%) | 0.009 | 0.056 |  | 0.001 | 0.040 |
| Radiotherapy (No *vs.* Yes) | 0.033 | 0.215 |  | 0.038 | 0.021 |
| Chemotherapy (No *vs.* Yes) | 0.011 | 0.134 |  | 0.020 | 0.091 |
| Endocrine therapy (No *vs.* Yes) | < 0.001 | < 0.001 |  | 0.001 | < 0.001 |
| Group (HER2-neg *vs.* HER2-pos-T) | 0.422 | 0.557 |  | 0.292 | 0.249 |

HER2, human epidermal growth factor receptor 2; BCS, breast-conserving surgery; IDC, invasive ductal carcinoma; ALN, axillary lymph node; LVI, lymph-vascular invasion; ER, estrogen receptor; PR, progesterone receptor; y/o, years old.





**Supplementary Fig. S1**

Cox proportional hazard analysis of DFS according to different subgroups.

HER2, human epidermal growth factor receptor 2; IDC, invasive ductal carcinoma; ALN: axillary lymph node; LVI, lymph-vascular invasion; ER, estrogen receptor; PR, progesterone receptor; BCS, breast-conserving surgery; HR, hazard ratio; y/o, years old.

**

Supplementary Fig. S2**

Kaplan-Meier estimates of DFS and OS for patients receiving neo-adjuvant therapy (a, b), chemotherapy (c, d) and radiotherapy (e, f). a. The 5-year DFS rate was 71.9% for the HER2-pos-T group and 71.3% for the HER2-neg group (*P* = 0.463). b. The 5-year OS rate was 87.7% and 85.1% for the two arms, respectively (*P* = 0.341). c. The estimated 5-year DFS rate was 89.3% for HER2- patients and 90.7% for HER2+ patients (*P* = 0.292). d. The estimated 5-year OS rate was 95.0% in the HER2-neg group and 96.0% in the HER2-pos-T group (*P* = 0.249). e. The 5-year DFS rate was 89.8% for the HER2-pos-T group and 87.9% for the HER2-neg group (*P* = 0.216). f. The 5-year OS rate was 96.6% and 95.4% for the two arms, respectively (*P* = 0.127).





**Supplementary Fig. S3**

Kaplan-Meier estimates of DFS and OS for patients receiving SERM (a, b), SERM-AI (c, d) and AI (e, f). a. The 5-year DFS rate was 89.7% for the HER2-pos-T group and 91.5% for the HER2-neg group (*P* = 0.573). b. The 5-year OS rate was 96.8% and 97.7% for the two arms, respectively (*P* = 0.651). c. The estimated 5-year DFS rate was 95.0% for HER2- patients and 95.2% for HER2+ patients (*P* = 0.605). d. The estimated 5-year OS rate was 99.1% in the HER2-neg group and 97.2% in the HER2-pos-T group (*P* = 0.228). e. The 5-year DFS rate was 90.0% for the HER2-pos-T group and 89.1% for the HER2-neg group (*P* = 0.334). f. The 5-year OS rate was 97.7% and 95.3% for the two arms, respectively (*P* = 0.042).


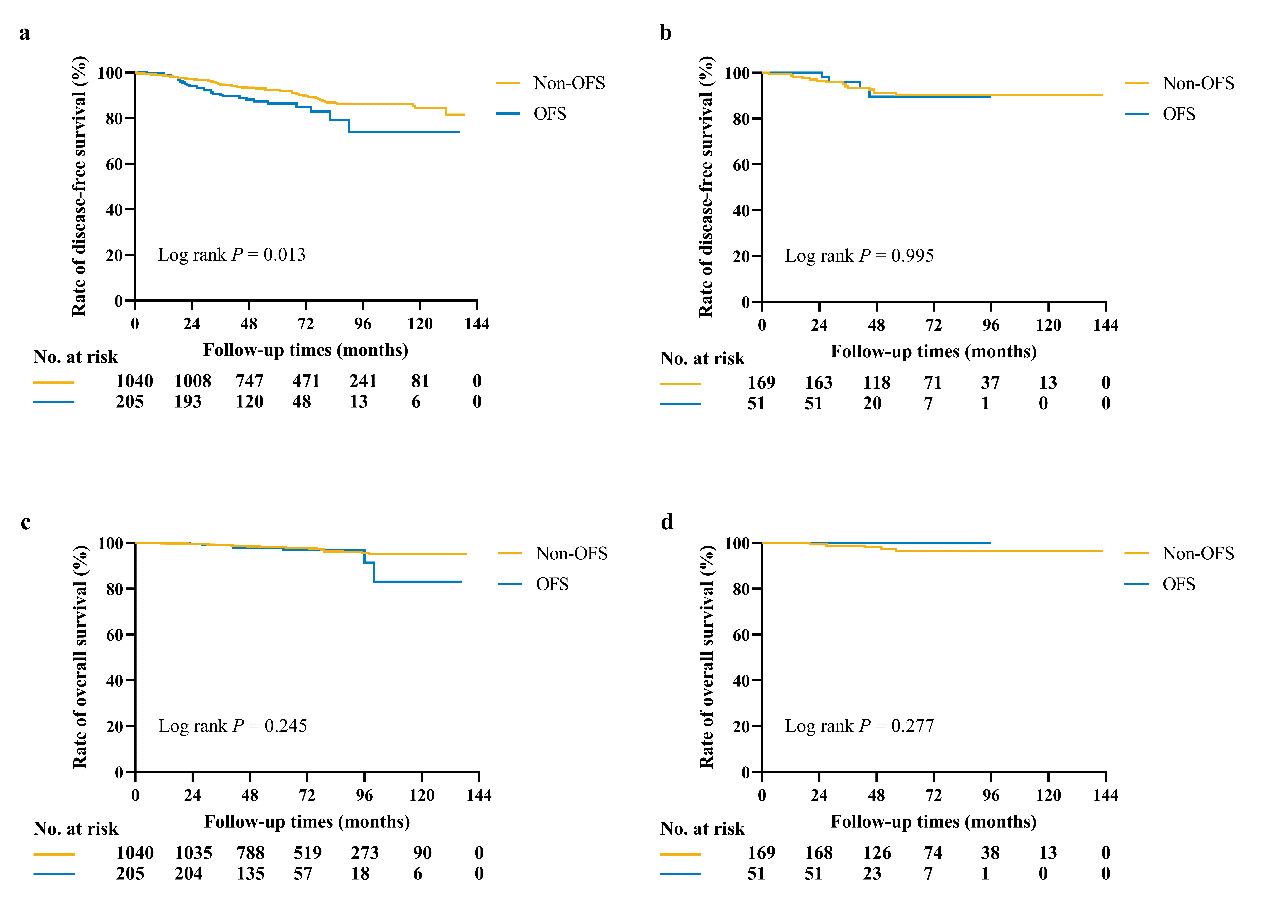


**Supplementary Fig. S4**

Kaplan-Meier estimates of DFS and OS comparing OFS vs. Non-OFS in HER2-neg group (a, c) and HER2-pos-T group (b, d). a. The 5-year DFS rate was 86.5% in the OFS arm and 92.2% in the Non-OFS group (*P* = 0.013). b. The 5-year DFS rate was 89.5% in the OFS arm and 90.2% in the Non-OFS group (*P* = 0.995). c. The 5-year OS rate was 97.8% in the OFS arm and 98.0% in the Non-OFS arm (*P* = 0.245). d. The 5-year OS rate was 100.0% in the OFS arm and 96.3% in the Non-OFS arm (*P* = 0.277).
